# Feasibility and Acceptability of a Mobile App and Wearable Device for Collecting Mental Health Survey and Passively Sensed Data Among Health Care Workers in Kenya: Mixed Methods Pilot Study

**DOI:** 10.2196/77761

**Published:** 2026-06-12

**Authors:** Linda Khakali, Andrew Aballa, Dorcas G Mwigereri, Eileen M Weinheimer-Haus, Willie Njoroge, Rachel Maina, Amos Bunde, Peris Musitia, Moses K Nyongesa, James Orwa, Jasmit Shah, Zhenke Wu, Anthony K Ngugi, Lukoye Atwoli, Srijan Sen, Candace Kolars, Akbar K Waljee, Amina Abubakar, Elena Frank, Zul Merali

**Affiliations:** 1 Brain and Mind Institute, Aga Khan University Nairobi, 3rd Parklands Avenue, Nairobi, 30270-00100, Kenya, 254 0704250183 ext 1629; 2 Department of Population Health, Aga Khan University Nairobi, Nairobi, Kenya; 3 Center for Global Health Equity, University of Michigan, Ann Arbor, MI, United States; 4 Department of Learning Health Sciences, University of Michigan Medical School, Ann Arbor, MI, United States; 5 Computing and Data Innovation Office, Aga Khan University Nairobi, Nairobi, Kenya; 6 Institute for Human Development, Aga Khan University Nairobi, Nairobi, Kenya; 7 Department of Biostatistics, University of Michigan, Ann Arbor, MI, United States; 8 Department of Medicine, Medical College East Africa, Aga Khan University Nairobi, Nairobi, Kenya; 9 Michigan Neuroscience Institute, University of Michigan, Ann Arbor, MI, United States; 10 Neurosciences Unit, Kenya Medical Research Institute, Wellcome Trust Research Programme, Kilifi, Kenya

**Keywords:** mental health, health care workers, mHealth, wearable device, mobile app, Kenya, low- and middle-income countries, Africa, mobile health

## Abstract

**Background:**

Mobile apps and wearable devices may help to facilitate early detection of mental health conditions by providing objective, real-time data to supplement other forms of feedback and diagnoses. Few studies have investigated the acceptability and feasibility of using a mobile app to track survey- and wearable-based data in mental health research in Sub-Saharan Africa.

**Objective:**

This pilot study evaluated the feasibility and acceptability of using a mobile app and wearables to capture mental health–based survey data and passively sensed data among Kenyan health care workers.

**Methods:**

A mixed methods study was conducted among health care workers employed at 4 hospitals in Nairobi, Kenya, over 30 days. A mobile app was used to collect and integrate active (baseline questionnaire and daily mood) and passive (wearable) data. The baseline questionnaire gathered information on sociodemographics, work environment, and mental health assessments on depression, anxiety, personality, early family environment, posttraumatic stress disorder, and substance use. A wearable device was used to gather data on steps, heart rate, and sleep. Qualitative interviews were conducted post trial to gain in-depth insights into participants’ experiences during the study.

**Results:**

Fifty-one participants enrolled in the pilot study. They were primarily nurses (47%) and female (70%), with a median (IQR) age of 32 (29-36) years. Attrition over 30 days was low, with only one participant dropping out due to device malfunction, which was a broken screen. Completeness of the baseline survey was high, with participants completing 96.1% of the questions. Further, 58% of the daily mood ratings were completed over the 30 days. For the wearable measures, participants submitted steps, heart rate, and sleep data on 93%, 73%, and 51% of study days, respectively. The proportion of days the wearable was worn for over 10 hours was 63%. Interviews revealed 2 primary themes. The first was intrinsic and extrinsic motivation; participants indicated that they liked having their health metrics tracked and receiving congratulatory messages from the app, encouraging increased step counts. The second theme was technical and usability challenges; 48% (10/21) of the participants reported discomfort wearing the watch while sleeping and challenges with synchronization of data due to the nonautomated nature of the process. Participants suggested additional prompts to remind them to complete the daily mood question.

**Conclusions:**

This pilot study demonstrates the feasibility of deploying mental health surveys, collecting data through wearable devices, and integrating such data within a single mobile platform under real-world infrastructure constraints. Health care workers in Kenya were willing to provide sensitive information through mental health assessments using a mobile app. To improve adherence, future studies should consider addressing some contextual factors such as daily prompts, enhanced data synchronization methods, and comfort concerns to improve adherence, especially during sleep.

## Introduction

Globally, health care workers (HCWs) play a crucial role in the health care system, in support of a wide array of responsibilities from patient care to supporting critical operational functions [1]. Human resources are limited in health care systems located in low- and middle-income countries (LMICs), resulting in strain on the professionals in the system [2]. Additionally, the nature of the work involves exposure to potentially traumatic events which may place HCWs at greater risk for psychological distress [3]. A meta-analysis including HCWs from 50 countries found that 42% reported anxiety symptoms, 33% reported depressive symptoms, and 42% reported insomnia, highlighting the high burden of these mental disorders [4]. In Kenya, similar findings were reported showing HCWs to be at risk of mental disorders such as depression at 32%, anxiety at 36%, and harmful alcohol use at 43.9%, and this was exacerbated during the COVID pandemic [5,6].

Prevention and early treatment of mental health conditions and psychological distress have the potential to reduce the impact of these disorders on the workforce and improve productivity and quality of output [7]. The traditional way of diagnosing mental health conditions is through questionnaires and scorecards during a patient visit [8]. However, point-in-time (eg, during an appointment with a health care provider) and retrospective questionnaires can result in the loss of valuable data informing diagnostic conclusions [9]. There is a growing interest in the use of digital technologies, such as smartphone apps and wearables, to augment traditional mental health care, particularly in LMICs where resources are limited for surveillance, diagnosis, and treatment. These approaches leverage streams of passively collected data captured from personal digital sensors to infer, monitor, and predict changes in health states over time [10-12].

Wearable devices capture objective indicators of sleep, physical activity, and heart rate, and changes in these variables have been closely linked to mental health states. For example, short or fragmented sleep has been associated with depression and anxiety, reduced physical activity has been linked to depressive symptoms, and elevated resting heart rate or reduced heart rate variability can reflect autonomic dysregulation observed in stress-related mood disorders [13-18]. Together, these measures provide real-time, ecological data that complement self-reported questionnaires and supplement longitudinal monitoring of mental health trajectories.

Wearable devices and mobile health (mHealth) technologies have been used in high-income countries to assess mental health indicators in various populations, including medical residents [18]. However, the integration of wearable device information with traditional questionnaires into a data collection platform in Africa has been scant, partly due to the infrastructure cost and limited availability of devices [19]. Despite these limitations, the continued increase in mobile phone access in LMICs such as Kenya provides an opportunity to digitize data collection through mobile apps [20]. In Kenya, studies have been conducted on the use of wearable devices for different health conditions and climate factors, with limited studies focused on mental health factors [21]. Thus, understanding the feasibility and acceptability of these platforms is important in assessing readiness for deployment of digitized platforms for mental health research in countries such as Kenya.

This pilot study evaluated the feasibility and acceptability of using a mobile technology app and wearables to collect both survey-based and passively sensed data among Kenyan HCWs to inform the deployment of a larger mHealth study. The primary focus was on the practical ability to deploy surveys, sustain device wear, and synchronize wearable data through an integrated app under real-world infrastructure constraints.

## Methods

### Research Design

This study used a sequential explanatory mixed methods approach where we conducted quantitative survey and wearable data collection for 30 days, after which qualitative interviews were completed to provide context to the quantitative outcomes. Our results are reported in line with the CONSORT (Consolidated Standards of Reporting Trials) extension for randomized pilot and feasibility trials (Multimedia Appendix 1) [22].

### Study Participants and Sampling Methods

HCWs, including nurses, doctors, psychologists, pharmacists, radiographers, physiotherapists, and nutritionists, were recruited from 4 urban health care facilities in Nairobi, Kenya from July to August 2022 (Table 1). Study inclusion criteria were aged 18 years or older, employment as an HCW, and smartphone ownership.

**Table 1.** Description of health care facilities in Nairobi, Kenya^a^^,b^.

Facility	Description
1	A level 5 tertiary private health care facility with 1088 health care workers.
2	A level 6 national referral hospital with 665 health care workers.
3	A level 5 county referral hospital, with 700 health care workers.
4	A level 5 obstetric and delivery referral hospital, with 396 health care workers.

a Health care facilities associated with a 30-day mixed methods pilot study focused on the feasibility and acceptability of using a mobile app to track survey- and wearable-based data in mental health research among Kenyan health care workers.

b Level 5 facilities are secondary referral hospitals that offer diagnostic and specialized care and more comprehensive medical care. Level 6 facilities are national referral and teaching hospitals.

### Recruitment

Recruitment strategies have been previously described [23]. Briefly, in-person sensitization meetings were conducted in study sites. During these sessions, HCWs were informed about the study objectives, duration, and study procedures. Additional recruitment strategies included email blasts, posters, and pamphlets placed around the health care facilities, and snowballing through participants who had already registered. Those interested in participating were invited to complete an online registration form that collected names, work cadre, email addresses, and phone numbers for enrollment. To increase reach, the registration form, along with brochures containing frequently asked questions sheets and QR codes for registering, was circulated in departmental WhatsApp (Meta Platforms) groups, Continuous Medical Education meetings, and virtual sensitization meetings. In total, 356 HCWs expressed interest in the study.

Of these, 60 individuals were randomly selected and stratified by facility using a random number generator. The target sample size for the pilot was n=50; thus, 60 individuals were contacted with an anticipated participation rate of 80%. The randomly selected individuals were contacted by email and invited to enroll in the pilot study within 7 days. Participants enrolled using the MyDataHelps (CareEvolution) mobile app. Within a week, 51 individuals provided informed consent and were enrolled in the study. Qualitative exit interviews were performed at the end of the study, with a subset of participants (n=22) selected through convenience sampling. Participants were recruited from individuals who were readily available and had participated in the broader study, rather than being selected through random sampling. Potential participants were identified based on their availability and willingness to participate in follow-up qualitative interviews. Representation across professional cadres and institutions was desired; however, the availability of participants was a key factor for inclusion due to demanding schedules. Participants were contacted by phone to schedule an appointment to meet face-to-face with the researchers to review the consent form. Of the 22 who were contacted, 1 participant declined to participate in the interviews.

### Survey and Wearable Data Collection Procedures

Following study enrollment, participants completed a baseline questionnaire that included information on sociodemographics, work environment, and the following assessments: (1) the Neuroticism, Extraversion, Openness to Experience Five-Factor Inventory to assess domains of personality in 5 areas [24]; (2) Risky Families Questionnaire to assess the degree of physical, mental, and emotional distress experienced in their homes during childhood and adolescence [25]; (3) Generalized Anxiety Disorder-7 to measure anxiety scores [26]; (4) Patient Health Questionnaire-9 to assess symptoms of depression [27]; (5) posttraumatic stress disorder (PTSD) checklist to assess presence and severity of PTSD symptoms [28]; and (6) World Health Organization Alcohol, Smoking and Substance Involvement Screening Test to assess substance use–related health risks and substance use disorders [29].

Following submission of the baseline questionnaire, participants received a Fitbit Inspire 2 (Fitbit Inc) and were instructed to wear the device continuously for the duration of the 30-day study. Participants were instructed on how to use wrist mode, verify that the sleep feature was on, and ensure syncing of the data with the mobile apps. Additionally, participants were advised to charge the device as soon as a low battery notification appeared. The battery life on Fitbit is up to 10 days on a single charge and charges to full in about 1 to 2 hours. Sleep, step count, heart rate, and mood rating were collected daily for 30 days.

The mobile app, MyDataHelps, was used to collect and integrate active (baseline questionnaire and daily mood) and passive (wearable) data. The app was available on Android and iOS operating systems, and on the web. Step-by-step instructions were provided for the web-based baseline questionnaire. The app provided a daily prompt to complete the mood rating (scale of 1-10). Wearable data were uploaded daily to the mobile app and integrated into the data collection platform through automatic synchronization from the Fitbit device to the Fitbit app on the participant’s mobile phone. To ensure the data synchronization from the Fitbit app to the MyDataHelps app, participants were required to have their mobile phone connected to the internet and Bluetooth enabled. The data synchronization process retrieved data stored on the device for up to 7 days. When gaps in data upload exceeded 3 days, the app sent a prompt to remind participants to synchronize their data.

### Exit Interviews

At the end of the 30-day pilot study period, a subset of participants was interviewed by members of the research team (LK, WN, and A Aballa) at the participants’ respective health facilities. The interview guide consisted of questions on user experience, challenges, and recommendations regarding the content and technology.

The interviews were conducted by trained social scientists. Prior to the interview, the interviewers completed a 3-day training on research ethics and the interview guide. A pretest was conducted with HCWs who were not part of the study, and the interview guide was later revised to ensure the flow and clarity of the questions. The interviews took approximately 40 to 70 minutes.

### Data Analysis

Feasibility of the pilot study was evaluated using baseline questionnaire response time, data completeness, recruitment results, and study attrition. For the baseline questionnaire, data completeness was calculated based on the proportion of visible questions answered, which excludes branching questions. Data completeness was also analyzed based on the number of days participants completed the daily mood rating and uploaded any steps, heart rate (heart rate intraday count), and sleep (duration) data. The average daily step count was also reported, excluding days with zero step count. The number of days participants wore the wearable for at least 10 hours was calculated using heart rate intraday minute count, as studies have suggested this is the minimal wear time to indicate a full day of use [30]. Descriptive statistics were calculated using R (version 4.1.1; R Core Team), and summary statistics were presented as mean and SD or median and IQR for continuous data, and frequencies and percentages for categorical data.

Acceptability of the baseline questionnaire and wearables was based on user experiences provided in qualitative exit interviews.

The interviews were recorded and transcribed verbatim, and a deductive thematic analysis approach was used to analyze the data with the support of NVivo Software (version 12; QSR International). Interviewers created field notes to capture participants’ impressions and observations during the interviews. The analysis followed the key stages of thematic analysis: familiarization with the data, generating initial codes, searching for themes, reviewing and refining themes, and defining and naming themes [31]. In the initial phase, transcripts were read thoroughly, and data were coded line by line, with concepts and ideas identified. As the analysis progressed, codes were refined and grouped into potential themes, which were then reviewed and further developed to reflect deeper meanings. In the final phase, the themes were clearly defined and organized to capture the core ideas of the study based on the objectives of the study. The coding matrices and final themes were reviewed and refined by LK, A Aballa, WN, and RM to ensure the accuracy and depth of the analysis.

### Ethical Considerations

The study obtained approvals from Aga Khan University Institutional Scientific Ethics and Review Committee (reference number 2021/IERC-166(V2)), the University of Nairobi and the Kenyatta National Hospital Ethics Review Committee (reference number KNH-ERC/A/473), regulatory bodies including the National Commission for Science Technology and Innovation (778557) and the Nairobi County and Nairobi Metropolitan Services (SMS; reference number EOP/NMS/HS/167), and hospital administrators in each study site. The study was conducted in accordance with the Declaration of Helsinki and written and informed consent was obtained from all participants. The consent form provided information on the study objective, willingness to participate, data safety, and confidentiality. For qualitative interviews, the consent included exit interview procedures and consent to be audio recorded. All participant data were deidentified using study identifier and not individual names, and data were securely transferred to the institution server. Due to budget constraints in maintaining prepaid data packages in LMICs, participants were compensated (KSh 500 or US $3.58 equivalent) for the purchase of prepaid data packages that were needed to support timely synchronization. Qualitative participants were compensated (KSh 1000 or US $7.06 equivalent) for their time. All participants were able to keep the wearable device at the end of the study.

## Results

### Participant Characteristics

**Figure 1.**
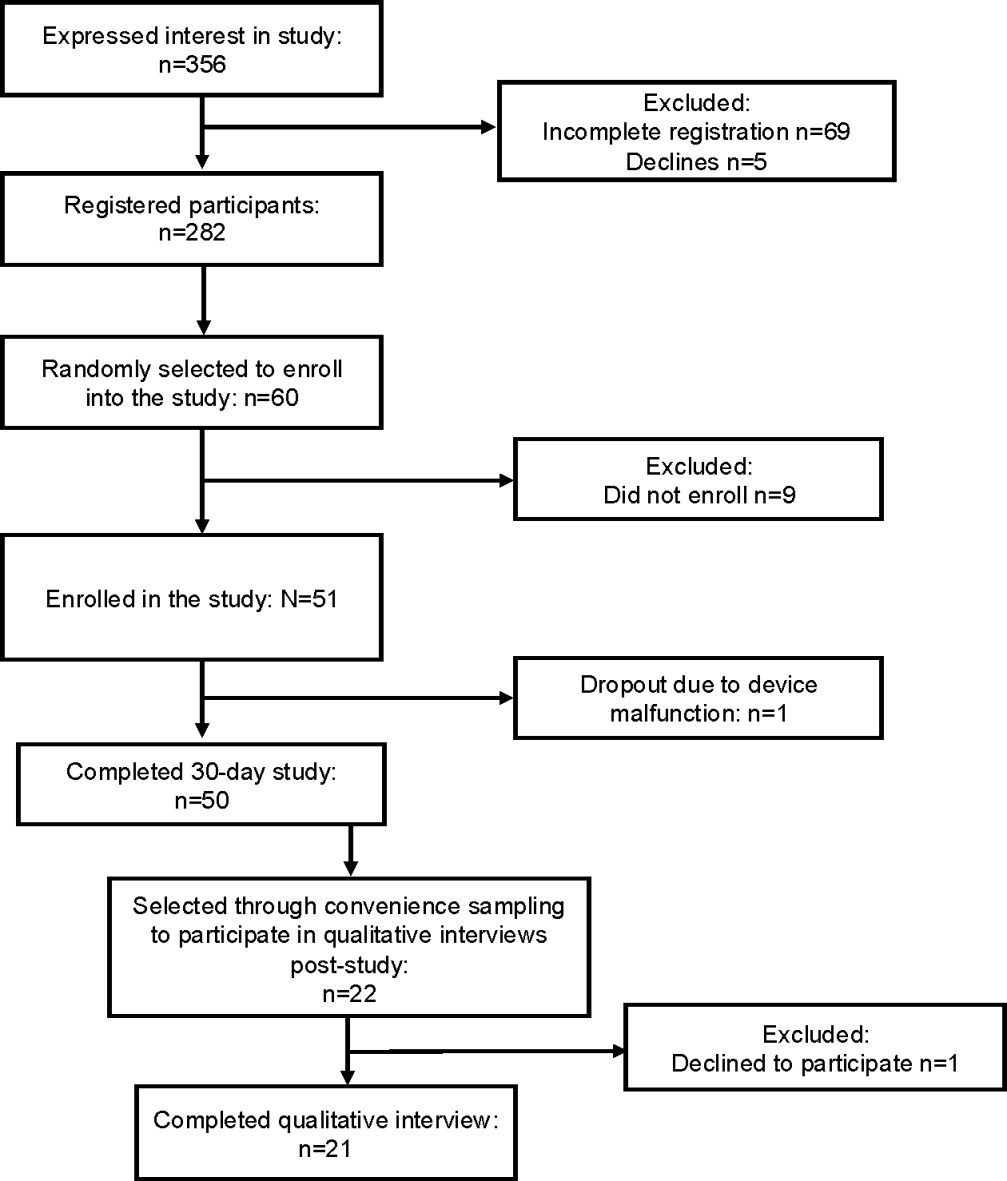
Enrollment flow of participants. Health care workers participated in a 30-day mixed methods pilot study to assess the feasibility and acceptability of using a mobile app and wearables to capture survey-based and passively sensed data in Kenya.

The participants were primarily female (70%), with 69% reporting attainment of a bachelor’s or master’s degree (Table 2). The median age of the participants was 32 (IQR 29‐36) years, and nurses (47%) were the largest health care cadre. The median duration of employment in health care was 8 (IQR 5‐12) years. The retention rate for the 30-day study duration was 98% (n=50). Characteristics of those who participated in the qualitative interviews are in Table 2 and Figure 1.

**Table 2.** Characteristics of enrolled participants and of those who subsequently took part in an exit interviews^a^.

Characteristic	All participants (N=51)	Exit interview participants (n=21)
Age (years), median (IQR)	32 (29‐36)	33 (28‐37)
Sex, n (%)		
Male	15 (30.0)	8 (38.1)
Female	36 (70.6)	13 (61.9)
Cadre, n (%)		
Doctors	10 (19.6)	1 (4.8)
Nurses	24 (47.1)	12 (57.1)
Others^b^	17 (33.3)	8 (38.1)
Years working in health care, median (IQR)	8 (5-12)	9 (7-13)
Sexual orientation, n (%)		
Heterosexual	48 (94.1)	20 (95.2)
Other or prefer not to say	3 (5.9)	1 (4.8)
Marital status, n (%)		
Not in a committed relationship	12 (23.5)	7 (35.0)
In a committed relationship	11 (21.6)	1 (5.0)
Married	26 (51.0)	12 (60.0)
Separated, divorced, or widowed	1 (2.0)	0 (0)
Highest education		
Diploma	16 (25.5)	8 (38.1)
Bachelor’s degree	25 (49.0)	10 (47.6)
Master’s degree	7 (13.7)	3 (14.3)
Other^b^	6 (11.8)	0 (0)
Residence		
Rural	1 (2.0)	1 (4.8)
Suburban	4 (7.8)	0
Urban	45 (88.2)	20 (95.2)
Prefer not to say	1 (2.0)	0
Facility		
1	18 (35)	6 (29)
2	15 (29)	9 (43)
3	10 (20)	3 (14)
4	8 (16)	3 (14)

a Participants enrolled in a 30-day mixed methods pilot study focused on the feasibility and acceptability of using a mobile application to track survey- and wearable-based data in mental health research among Kenyan health care workers.

b Nutritionists, pharmacists, physiotherapists, and psychologists.

### Feasibility of Mobile App and Wearable for Data Collection

The median time to complete the baseline questionnaire was 1 (IQR 1‐2, range 1-11) day, from the date the link was received to submission. All participants (N=51) completed 100% of questions in the Risky Families Questionnaire, Generalized Anxiety Disorder-7, Patient Health Questionnaire-9, and PTSD assessments, while 92% (n=47) and 84.4% (n=42) completed all questions in the Neuroticism, Extraversion, Openness to experience Five-Factor Inventory and World Health Organization Alcohol, Smoking and Substance Involvement Screening Test assessment, respectively.

The wearable was worn for >10 hours on 63% of study days (n=50). Nearly all participants uploaded step (n=50), heart rate (n=46), and sleep (n=45) data within 5 days of receiving the wearable. On average, participants completed 58.4% of the daily mood questions over the 30 days. Data on steps, heart rate, and sleep were uploaded on 92.6%, 76.6%, and 49.8% of the days, respectively. The average number of daily steps was 8180 (SD 4872).

To further characterize variability in adherence, we examined the median (IQR) and distribution of the number of days each participant submitted data (Table 3 and Multimedia Appendix 1). Approximately half of the participants submitted heart rate (n=26) and step count (n=31) data on all 30 study days, indicating higher adherence for these 2 passively collected metrics (Multimedia Appendix 1). In contrast, submissions for mood and sleep and the number of days with >10 hours of data (ie, wear time) were more variable across participants and across days. Histograms of the number of submission days show a more dispersed pattern for these measures; however, the distributions were not strongly skewed toward a small subset of highly adherent individuals.

**Table 3.** Completeness of longitudinal data (n=50)^a^^,b^.

Variable	Days, median (IQR)
Wear time >10 hours	21 (13‐28)
Steps	30 (28‐30)
Heart rate	30 (18‐30)
Sleep	15 (7‐23)
Mood	18 (13‐23)

a Participants enrolled in a 30-day mixed methods pilot study focused on the feasibility and acceptability of using a mobile app to track survey- and wearable-based data in mental health research among Kenyan health care workers.

b Completeness was based on the average number of days over the 30-day pilot that participants wore the wearable for >10 hours; uploaded any step, heart rate, and sleep data; and completed the daily mood question.

### Acceptability of Mobile App and Wearable for Data Collection

#### Theme 1: Experiences With Study Protocols

Qualitative interviews with participants on baseline questionnaires and protocols elicited both negative and positive experiences. The participants had mixed perceptions of the administration of the baseline questionnaire. The positive experience was attributed to the questionnaire’s simple language, questions interlinked, making it easy to follow through, and flexible to complete at one’s convenience due to the online format, while the challenge was attributed to the questionnaire being lengthy and challenging to complete in one sitting.

The questions were simplified so they were easy to understand and of course relate... the questions tied to the study.

[Pharmacist]

I think the baseline survey was so long. I didn’t finish at once. I’m guessing maybe I started when I was a bit busy, but it was long. I almost gave up.

[Facility 1, male medical officer]

#### Theme 2: Intrinsic and Extrinsic Motivation

Participants expressed that the wearable and MyDataHelps app significantly increased their awareness of personal health behaviors, such as steps taken, water intake (which was an optional feature), and sleep patterns. The wearable had additional metrics that could be activated by participants, but the study focused on heart rate, sleep, and activity rate. The wearable device encouraged goal setting and helped track progress, providing users with regular reminders and alerts to stay on track.

It has come in handy in terms of reminding me how many steps I have done in a day... it updates me when to take my water.

[Physiotherapist]

It actually made me cautious about my sleep patterns and sleep hygiene.

[Clinical officer]

Congratulatory messages and goal reminders from the app positively affected participants’ motivation. Many felt encouraged by progress notifications, which fostered a sense of accomplishment and supported mental wellness. This motivation extended to family and social contexts, as some participants began to actively engage in physical activities with family members.

I can walk, and it tells me congratulations you’ve done this many during the day apart from checking my mood, it’s also checking my health.

[Nurse]

Additionally, the app was reported to facilitate self-monitoring, primarily through mood-tracking and daily health prompts. Participants accepted the mobile app because of its ability to track their mood at the end of each day, which helped them reflect on their mental health.

This feature encouraged daily self-reflection, allowing users to evaluate their day and develop an awareness of patterns in their mood.

[Nurse]

The wearable was acceptable based on features such as water resistance, as well as the ability to keep power for a long time. The straps were found to be light and easy to wear throughout the day and night. These features were encouraging for participants to have the wearable on throughout the day and night with ease and comfort.

The good thing is it keeps power very well. I charge maybe once in every two weeks.

[Nurse]

When I realized it had a function of water lock then I decided that I will not be removing it at all, so when I’m going to shower, I activate water lock, so I have it all the time.

[Nurse]

#### Theme 3: Technical and Usability Challenges

Despite the value of wearable devices, 48% (10/21) of the participants reported challenges with wearable design and technical requirements. Continuous Bluetooth connectivity was necessary for data syncing, which participants found inconvenient, especially when forgetting to activate Bluetooth for extended periods, sometimes leading to data collection gaps.

The only thing is that if you’re not online or not connected with Bluetooth, you might not be able to enter data.

[Nurse]

Participants recommended providing additional reminders and motivational prompts to ensure adherence to the study. Two participants expressed a desire for more color varieties for the straps and to receive a replacement if the wearable broke, as replacements were not provided in this pilot.

Most of the participants noted some discomfort sleeping with the wearable on their wrist to record their sleep patterns in the initial days, but this improved with time.

It felt uncomfortable, you had had the watch the whole day, and sometimes at night, I would try but would wake up and remove it(watch) because it just felt uncomfortable.

[Nurse]

Then being plastic sometimes just gets sort of uncomfortable. So, you’d want to change from one hand to another.

[Nurse]

Participants encountered technical challenges with the mobile app, primarily regarding the requirement for consistent internet and Bluetooth connectivity. The app required participants to log in daily at a set time, but limited internet data constrained consistent access, which could delay synchronization of data to the mobile app or lead to a missed daily prompt. Participants struggled with the Bluetooth connectivity for synchronization of data and wished that the app could only work with internet access to synchronize.

It’s only that it won't synchronize data when either your battery is low.

[Nurse]

The only thing is that if you’re not online or not connected with Bluetooth, you might not be able to enter data

[Nurse]

Recommendations were provided on simplifying the app’s connectivity requirements to reduce the dependency on Bluetooth and facilitate prepaid mobile data packages. Suggestions included the possibility of offline functionality for data logging, which could sync automatically when internet access is restored.

Bluetooth is necessary for pairing, but I wish it was not required. Just the bundle would have been enough because most of the time you realize you are online on your phone but using Bluetooth is a habit that most of us do not have.

[Clinical officer]

## Discussion

### Principal Findings

This pilot study supports the feasibility of deploying mental health surveys, collecting data through wearables, and integrating wearable and survey data within a single mobile platform under real-world infrastructure constraints among Kenyan HCWs. Participant retention and completion of the initial questionnaire were high, indicating strong initial engagement and comfort reporting on mental health symptoms. Daily adherence was moderate; approximately half of the participants submitted heart rate and step count data on all 30 study days, while mood, steps, and wear time (≥10 h/d) were more variable across participants and over time. Sleep data were only captured on about half of all study days. Together, these metrics suggest that while continuous daily engagement was not fully achieved, the high retention and collection of multidimensional data support that these technologies are logistically viable in similar LMIC settings. Moreover, participants reported largely positive experiences with the platform, especially appreciating the self-monitoring aspect and feedback features, further supporting the feasibility and acceptability of this approach.

Mental health research in HCWs is critical to identifying and reducing stress, burnout, and psychological harm in those who care for others, which in turn improves worker well-being and patient care. This pilot study revealed that HCWs were willing to provide sensitive mental health information, such as depression, anxiety, posttraumatic stress disorder, and substance use, within a day of enrollment. Studies in Africa have highlighted that the absence of native terminology for mental health and the existence of unwritten nondisclosure norms make it difficult to openly initiate conversations about mental health [32]. Willingness to provide sensitive information on mental health is a critical step in identifying people at risk for mental health disorders. In this pilot, the web-based mode of administration and the preenrollment sensitization process, which helped build rapport between the study team and participants while addressing their concerns and questions about data confidentiality, may have mitigated some stigma-related barriers to disclosure as other studies have suggested [33,34].

The use of wearables in mental health research to infer, monitor, and predict changes in health states over time is an emerging area. On average, studies that have used wearables to collect data among HCWs with small sample sizes or shorter duration of data collection have reported a retention rate of 83%‐98% [35,36], which aligns with the retention rate in this pilot. In our study, participants reported that motivation to know their health information was a contributor to high retention, and other studies have shown motivational support to be a contributor in reducing attrition [37]. In addition, all 51 participants completed the baseline questionnaire, a rate that was 2 times higher than a similar study conducted in the United States where the completion rate was 48% [38]. The differences could be due to the varied recruitment approaches. Unlike the US-based study that used a mainly monetary incentive-driven approach to recruit participants, our study used a multipronged recruitment approach, including increased physical contact through sensitization, where we had one-on-one discussions with participants as opposed to sharing information via email or website, which could be more impersonal reimbursement for internet connectivity, email, and phone notifications. Similar studies have reported that adopting such a comprehensive approach during recruitment of participants for web-based surveys leads to better engagement [39].

In terms of adherence to daily data collection, our findings are consistent with results from similar digital health studies in both low- and high-income settings. For example, a 5-day pilot in Uganda reported high compliance with wearable use and daily diary completion, despite challenges with comfort during sleep [40]. Similarly, a 3-week study in rural Kenya demonstrated high step and sleep data completeness, but limited heart rate data availability [41]. The wear time observed in our study (63% of days with over 10 h of usage) is within the typical range of 50%‐70% reported in free-living environments [30]. Additionally, mood surveys were completed on two-thirds of the days, which is encouraging given the known challenges of sustaining engagement in digital health monitoring. For example, the average 30-day retention rates for mobile apps in general are less than 6% [42,43]. Collectively, these findings highlight the feasibility of digital monitoring among HCWs in LMICs.

Several barriers likely contributed to moderate adherence to the daily data collection, particularly for sleep and daily mood entries. Participants cited discomfort wearing the device overnight, contributing to reduced sleep data availability. Similar issues have been documented in other wearable studies [40,41] and highlight the importance of comfort for extended wear. Technical challenges with the wearable included instances where the device was accidentally switched from wrist mode to clip mode, which turns off sleep monitoring. Participants were guided on how to check if the wearable was in wrist mode during the initial onboarding to minimize potential data loss. However, this is an important consideration for future studies that leverage wearable devices with the functionality to switch modes. Regarding daily mood, reporting may have been impacted by notification delivery failures. Push notifications relied on continuous internet access, which was not always available. Interestingly, studies reporting higher adherence to daily mood diaries have reported using SMS as opposed to push notifications [44], which may circumvent the need for Wi-Fi. Participants also reported forgetfulness, competing work demands, and survey fatigue, which are consistent with prior research indicating that survey delivery mode, timing, and user workload influence daily self-report adherence [40,45]

Despite these challenges, our findings suggest strong participant engagement. Qualitative feedback revealed largely positive perceptions about using the mobile app and wearable device. Many participants appreciated tracking their health metrics and receiving motivational messages (eg, congratulatory prompts for meeting step count goals), indicating that these features likely enhanced engagement. Future studies that use wearables in mental health research may need to consider that beyond serving as an assessment tool, the wearable itself may also influence participant behavior. Increased awareness of sleep patterns and physical activity may motivate behavior change, which may function as an unintended intervention and potentially confer mental health benefits.

### Limitations

The study was conducted on a small sample size, which is appropriate for a pilot study, but limits our ability to detect subgroup or temporal comparisons. In addition, the population was limited to 4 urban hospitals with an overrepresentation of nurses, which may limit the generalizability of the findings to rural areas and other cadres. In addition, the trial duration was only 1 month, which captures feasibility in the short term, but adherence patterns might change over longer periods. It remains unclear whether participants would continue using the app and wearable for an extended period of time (eg, months). Qualitative feedback was obtained from a subset of participants who completed the study; those who had more difficulties might have been less likely to participate in the interviews, potentially leading to a positive bias in qualitative results. Collectively, this pilot focused on the practical ability to deploy surveys, sustain device wear, and synchronize wearable data under real-world infrastructure constraints, rather than on deriving clinically interpretable mental health metrics such as heart rate variability or complete daily physical activity profiles, which were beyond the scope of this pilot. Future studies are warranted to better link wearable-derived measures with mental health outcomes and to evaluate clinically interpretable metrics in larger, more comprehensive studies. Despite these limitations, the study provides valuable preliminary evidence to inform the scaling of similar digital mental health studies in HCWs in LMICs.

### Conclusion

This pilot study provides justification for the use of a mobile app and wearable in mental health research in LMICs. The high survey completion rate and study retention, as well as qualitative acceptability, reflect strong engagement and suggest that such technologies are logistically viable in similar contexts. Future studies looking to scale similar approaches should consider enhancing prompts, improving device comfort, and ensuring reliable internet connectivity or finding an alternative solution.

## Supplementary material

10.2196/77761 Multimedia Appendix 1 Distributions of data completeness.

## Abbreviations

CONSORT: Consolidated Standards of Reporting Trials
HCW: health care worker
LMIC: low- and middle-income country
mHealth: mobile health
PTSD: posttraumatic stress disorder

## References

[1] Alzamanan AM, Almareh NF, Alyami AAM (2024). The role of healthcare workers in modern medicine: a comprehensive review of challenges, contributions, and future directions. J Ecohumanism.

[2] Ahmat A, Okoroafor SC, Kazanga I (2022). The health workforce status in the WHO African Region: findings of a cross-sectional study. BMJ Glob Health.

[3] Walton M, Murray E, Christian MD (2020). Mental health care for medical staff and affiliated healthcare workers during the COVID-19 pandemic. Eur Heart J Acute Cardiovasc Care.

[4] Aymerich C, Pedruzo B, Pérez JL (2022). COVID-19 pandemic effects on health worker’s mental health: systematic review and meta-analysis. Eur Psychiatry.

[5] Kwobah EK, Mwangi A, Patel K, Mwogi T, Kiptoo R, Atwoli L (2021). Mental disorders among health care workers at the early phase of COVID-19 pandemic in Kenya; findings of an online descriptive survey. Front Psychiatry.

[6] Jaguga F, Kwobah EK, Mwangi A (2022). Harmful alcohol use among healthcare workers at the beginning of the COVID-19 pandemic in Kenya. Front Psychiatry.

[7] Søvold LE, Naslund JA, Kousoulis AA (2021). Prioritizing the mental health and well-being of healthcare workers: an urgent global public health priority. Front Public Health.

[8] Roe D, Mazor Y, Gelkopf M (2022). Patient-reported outcome measurements (PROMs) and provider assessment in mental health: a systematic review of the context of implementation. Int J Qual Health Care.

[9] Fung CH, Hays RD (2008). Prospects and challenges in using patient-reported outcomes in clinical practice. Qual Life Res.

[10] Marconcin P, Werneck AO, Peralta M (2022). The association between physical activity and mental health during the first year of the COVID-19 pandemic: a systematic review. BMC Public Health.

[11] Stautland A, Jakobsen P, Fasmer OB (2023). Reduced heart rate variability during mania in a repeated naturalistic observational study. Front Psychiatry.

[12] Yang FN, Liu TT, Wang Z (2022). Functional connectome mediates the association between sleep disturbance and mental health in preadolescence: a longitudinal mediation study. Hum Brain Mapp.

[13] Daghlas I, Lane JM, Saxena R, Vetter C (2021). Genetically proxied diurnal preference, sleep timing, and risk of major depressive disorder. JAMA Psychiatry.

[14] Matcham F, Carr E, Meyer N (2024). The relationship between wearable-derived sleep features and relapse in major depressive disorder. J Affect Disord.

[15] Jacobson NC, Feng B (2022). Digital phenotyping of generalized anxiety disorder: using artificial intelligence to accurately predict symptom severity using wearable sensors in daily life. Transl Psychiatry.

[16] Zhang Y, Stewart C, Ranjan Y (2025). Large-scale digital phenotyping: Identifying depression and anxiety indicators in a general UK population with over 10,000 participants. J Affect Disord.

[17] Jo YT, Lee SW, Park S, Lee J (2024). Association between heart rate variability metrics from a smartwatch and self-reported depression and anxiety symptoms: a four-week longitudinal study. Front Psychiatry.

[18] Horwitz A, Czyz E, Al-Dajani N (2022). Utilizing daily mood diaries and wearable sensor data to predict depression and suicidal ideation among medical interns. J Affect Disord.

[19] Dai B, Larnyo E, Tetteh EA, Aboagye AK, Musah AAI (2020). Factors affecting caregivers’ acceptance of the use of wearable devices by patients with dementia: an extension of the unified theory of acceptance and use of technology model. Am J Alzheimers Dis Other Demen.

[20] Maree J, Piontak R, Omwansa T, Shinyekwa I, Njenga K (2013). Developmental uses of mobile phones in Kenya and Uganda. SSRN J.

[21] Barteit S, Boudo V, Ouedraogo A (2021). Feasibility, acceptability and validation of wearable devices for climate change and health research in the low-resource contexts of Burkina Faso and Kenya: study protocol. PLoS One.

[22] Thabane L, Hopewell S, Lancaster GA (2016). Erratum to: Methods and processes for development of a CONSORT extension for reporting pilot randomized controlled trials. Pilot Feasibility Stud.

[23] Aballa A, Mwigereri DG, Zhao Z (2026). Depressive symptoms and associated factors among Kenyan health care workers. JAMA Psychiatry.

[24] Murray G, Rawlings D, Allen NB, Trinder J (2003). NEO Five-Factor Inventory scores: psychometric properties in a community sample. Meas Eval Couns Dev.

[25] Taylor SE, Lerner JS, Sage RM (2011). Risky Families Questionnaire. PsycTESTS Dataset.

[26] Dhira TA, Rahman MA, Sarker AR, Mehareen J (2021). Validity and reliability of the Generalized Anxiety Disorder-7 (GAD-7) among university students of Bangladesh. PLoS One.

[27] Mwangi P, Nyongesa MK, Koot HM, Cuijpers P, Newton CRJC, Abubakar A (2020). Validation of a Swahili version of the 9-item Patient Health Questionnaire (PHQ-9) among adults living with HIV compared to a community sample from Kilifi, Kenya. J Affect Disord Rep.

[28] Bovin MJ, Marx BP, Weathers FW (2016). Psychometric properties of the PTSD Checklist for Diagnostic and Statistical Manual of Mental Disorders-Fifth Edition (PCL-5) in veterans. Psychol Assess.

[29] Humeniuk R, Henry-Edwards S, Ali R, Poznyak V, Monteiro MG (2010). The Alcohol, Smoking and Substance Involvement Screening Test (ASSIST): manual for use in primary care. World Health Organization.

[30] Chan A, Chan D, Lee H, Ng CC, Yeo AHL (2022). Reporting adherence, validity and physical activity measures of wearable activity trackers in medical research: a systematic review. Int J Med Inform.

[31] Braun V, Clarke V (2006). Using thematic analysis in psychology. Qual Res Psychol.

[32] Jumbe S, Nyali J, Simbeye M, Zakeyu N, Motshewa G, Pulapa SR (2022). “We do not talk about it”: engaging youth in Malawi to inform adaptation of a mental health literacy intervention. PLoS One.

[33] Bidonde J, Meneses-Echavez JF, Hafstad E, Brunborg GS, Bang L (2023). Methods, strategies, and incentives to increase response to mental health surveys among adolescents: a systematic review. BMC Med Res Methodol.

[34] Ericson A, Bonuck K, Green LA, Conry C, Martin JC, Carney PA (2023). Optimizing survey response rates in graduate medical education research studies. Fam Med.

[35] Mendelsohn D, Despot I, Gooderham PA, Singhal A, Redekop GJ, Toyota BD (2019). Impact of work hours and sleep on well-being and burnout for physicians-in-training: the Resident Activity Tracker Evaluation Study. Med Educ.

[36] Jevsevar DS, Molloy IB, Gitajn IL, Werth PM (2021). Orthopaedic surgeon physiological indicators of strain as measured by a wearable fitness device. J Am Acad Orthop Surg.

[37] Egilsson E, Bjarnason R, Njardvik U (2023). Usage and daily attrition of a smartphone-based health behavior intervention: randomized controlled trial. JMIR Mhealth Uhealth.

[38] Li SX, Halabi R, Selvarajan R (2022). Recruitment and retention in remote research: learnings from a large, decentralized real-world study. JMIR Form Res.

[39] Meyer VM, Benjamens S, Moumni ME, Lange JFM, Pol RA (2022). Global overview of response rates in patient and health care professional surveys in surgery: a systematic review. Ann Surg.

[40] Nielsen K, Mobley K, Culbreth R, Palmier J, Nabulya A, Swahn MH (2024). Feasibility and acceptability of wearable devices and daily diaries to assess sleep and other health indicators among young women in the slums of Kampala, Uganda. Digit Health.

[41] Matzke I, Huhn S, Koch M (2024). Assessment of heat exposure and health outcomes in rural populations of western Kenya by using wearable devices: observational case study. JMIR Mhealth Uhealth.

[42] Tafradzhiysk N Mobile app retention rates (2025). Business of Apps.

[43] Lipschitz JM, Pike CK, Hogan TP, Murphy SA, Burdick KE (2023). The engagement problem: a review of engagement with digital mental health interventions and recommendations for a path forward. Curr Treat Options Psychiatry.

[44] Kalmbach DA, Fang Y, Arnedt JT (2018). Effects of sleep, physical activity, and shift work on daily mood: a prospective mobile monitoring study of medical interns. J Gen Intern Med.

[45] Huhn S, Matzke I, Koch M (2022). Using wearable devices to generate real-world, individual-level data in rural, low-resource contexts in Burkina Faso, Africa: a case study. Front Public Health.

