# Supplementary material for: Feasibility and Acceptability of a Mobile App and Wearable Device for Collecting Mental Health Survey and Passively Sensed Data Among Health Care Workers in Kenya: Mixed Methods Pilot Study
Source: JMIR Mhealth Uhealth. 2026 Jun 12;14:e77761. doi: 10.2196/77761 (PMC13263018; doi:10.2196/77761)
Supplement: Multimedia Appendix 1 [file mhealth-v14-e77761-s001.docx]

**Multimedia Appendix 1**

**Figure S2. Distributions of data completeness.** Healthcare workers participated in a 30-day mixed methods pilot study to assess the feasibility and acceptability of using a mobile application and wearables to capture survey-based and passively sensed data in Kenya. n=50 were included in analysis of data completeness of the daily mood question and wearable data over the 30-day study period. Histograms depict the number of days participants completed the daily mood rating and uploaded any steps, heart rate (heart rate intraday count), and sleep (duration) data. The number of days participants wore the wearable for at least 10 hours was calculated using heart rate intraday minute count.

**
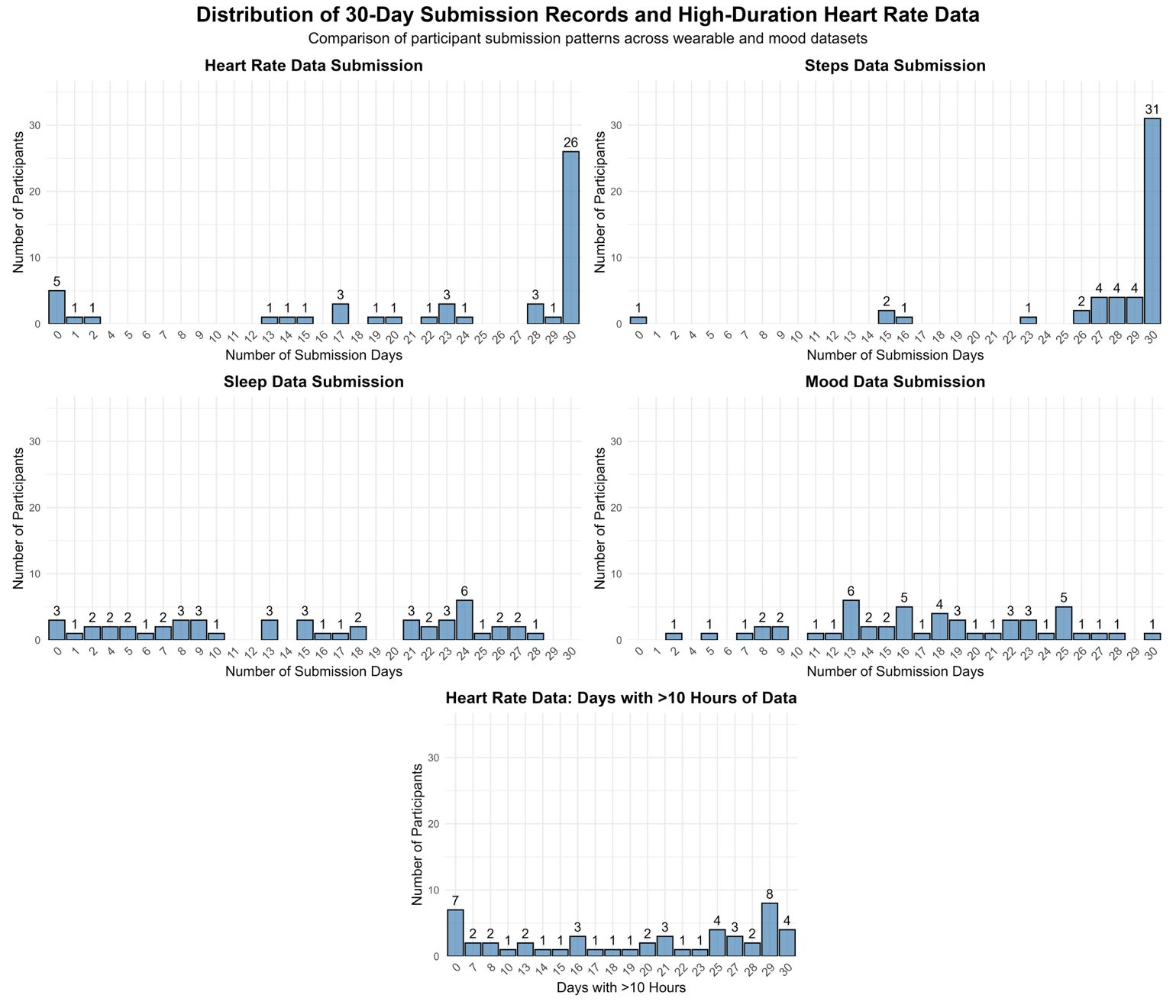
**
